# A detailed workflow to develop QIIME2-formatted reference databases for taxonomic analysis of DNA metabarcoding data

**DOI:** 10.1186/s12863-022-01067-5

**Published:** 2022-07-08

**Authors:** Benjamin Dubois, Frédéric Debode, Louis Hautier, Julie Hulin, Gilles San Martin, Alain Delvaux, Eric Janssen, Dominique Mingeot

**Affiliations:** 1 Life Sciences Department, Bioengineering Unit, Walloon Agricultural Research Center, Chaussée de Charleroi 234, 5030 Gembloux, Belgium; 2 Life Sciences Department, Plant and Forest Health Unit, Walloon Agricultural Research Center, Rue de Liroux 2, 5030 Gembloux, Belgium; 3 Knowledge and Valorization of Agricultural Products Department, Quality and Authentication Unit, Walloon Agricultural Research Center, Chaussée de Namur 24, 5030 Gembloux, Belgium; 4 Knowledge and Valorization of Agricultural Products Department, Protection, Control Products and Residues Unit, Walloon Agricultural Research Center, Rue du Bordia 11, 5030 Gembloux, Belgium

**Keywords:** Reference database, QIIME2, Bioinformatics workflow, Metabarcoding, High-throughput sequencing, ITS2, *rbcL*, Plant

## Abstract

**Background:**

The DNA metabarcoding approach has become one of the most used techniques to study the taxa composition of various sample types. To deal with the high amount of data generated by the high-throughput sequencing process, a bioinformatics workflow is required and the QIIME2 platform has emerged as one of the most reliable and commonly used. However, only some pre-formatted reference databases dedicated to a few barcode sequences are available to assign taxonomy. If users want to develop a new custom reference database, several bottlenecks still need to be addressed and a detailed procedure explaining how to develop and format such a database is currently missing. In consequence, this work is aimed at presenting a detailed workflow explaining from start to finish how to develop such a curated reference database for any barcode sequence.

**Results:**

We developed DB4Q2, a detailed workflow that allowed development of plant reference databases dedicated to ITS2 and *rbcL*, two commonly used barcode sequences in plant metabarcoding studies. This workflow addresses several of the main bottlenecks connected with the development of a curated reference database. The detailed and commented structure of DB4Q2 offers the possibility of developing reference databases even without extensive bioinformatics skills, and avoids ‘black box’ systems that are sometimes encountered. Some filtering steps have been included to discard presumably fungal and misidentified sequences. The flexible character of DB4Q2 allows several key sequence processing steps to be included or not, and downloading issues can be avoided. Benchmarking the databases developed using DB4Q2 revealed that they performed well compared to previously published reference datasets.

**Conclusion:**

This study presents DB4Q2, a detailed procedure to develop custom reference databases in order to carry out taxonomic analyses with QIIME2, but also with other bioinformatics platforms if desired. This work also provides ready-to-use plant ITS2 and *rbcL* databases for which the prediction accuracy has been assessed and compared to that of other published databases.

**Supplementary Information:**

The online version contains supplementary material available at 10.1186/s12863-022-01067-5.

## Background

Traditionally, the identification of plant species has been carried out through morphological identification and via microscopic examination. Despite being simple and cost-effective, these strategies rely on the experience of a few experts and the distinction between closely related specimens may not be possible. In addition, morphological identification relies on the analysis of tissues or even whole plants, which makes it inappropriate to study processed products. The development of molecular techniques has opened new possibilities for plant identification. Among them, DNA barcoding enables identification of individual specimens through the amplification and sequencing of one (or several) taxonomically informative DNA sequence, called barcode sequence [1, 2]. Recent advances in high-throughput sequencing (HTS) technologies brought this strategy to a new level, i.e. metabarcoding, by simultaneously DNA barcoding multiple species in complex samples [3]. DNA metabarcoding has already been widely used to assess the plant species composition of complex pollen samples [4, 5], study plant-pollinator interaction networks [6, 7], authenticate food products [8–10] and medicines [11], analyze plant components of human diets [12], investigate the belowground plant diversity [13] or even that found in an alpine glacier area [14].

To assess the sample taxa composition in such an approach, one of the key points is the availability of curated reference databases, which allow taxonomic assignment of sequencing reads to be carried out. Several works have already focused on the development of reference datasets dedicated to the internal transcribed spacer 2 (ITS2) and the ribulose-1,5-bisphosphate carboxylase-oxygenase (*rbcL*) markers, two barcode sequences used in this study. One of the first initiatives to develop an ITS2 reference database was carried out by Schultz and colleagues [15], initially as a data repository. The database was then updated several times – last update in 2015 by Ankenbrand et al. [16] – and its structure evolved to an interactive workbench. Sickel et al. then extracted all *Viridiplantae* sequences from this database to analyze plant metabarcoding data [17]. This reference library has then been used in several DNA metabarcoding studies [18–21]. A similar work was carried out in 2017 by Bell and her colleagues to build a reference database dedicated to the *rbcL* barcode [18]. This database was updated in 2021 [22] and, in the same study, Bell and co-authors also developed a new ITS2 database dedicated to flowering plants (i.e. the *Magnoliopsida* class). Another initiative has been led by Curd et al. in 2019 [23] with the CRUX database generation module. This module is part of the Anacapa toolkit, which also allows processing of HTS data, assigning taxonomy and exploring results. In 2020, Richardson and colleagues [24] developed MetaCurator, another toolkit to generate reference databases dedicated to taxonomically informative genetic markers. Banchi et al. developed in 2020 a set of databases called PLANiTS, which groups three reference datasets dedicated to the ITS regions (ITS1, ITS2 or the full ITS) [25]. Finally, the BCdatabaser tool was developed by Keller et al. in 2020 [26] and both command-line and web-interface formats are available. It allows linking sequence and taxonomic information retrieved from NCBI and formatting the output to be readable by current taxonomic classifiers.

Such reference databases must be used within a complete bioinformatics pipeline to deal with the huge amount of raw data generated by the HTS step. This allows processing of sequencing data, retaining only high quality reads and assigning them a taxonomy, among other things. Several bioinformatics platforms like QIIME1 [27], USEARCH [28], Mothur [29] and OBITools [30] are available to carry out end-to-end analysis of HTS data. Recently, QIIME2 [31] (https://qiime2.org/) has been developed and has become one of the most used bioinformatics platforms in recent metabarcoding studies [32–34]. QIIME2 is a plugin-based, community developed and open source bioinformatics platform dedicated to HTS data analysis, with a focus on data and analysis transparency. Indeed, it includes a unique system of data provenance tracking, ensuring reproducibility of the analysis by recording details of every bioinformatics step (i.e. commands called, arguments and parameters provided, information about the computational environment in which the analysis was carried out). QIIME2 has been included in several bioinformatics pipeline-benchmarking analyses. Several studies showed that performances of QIIME2 meet or often exceed those of other platforms to which it was compared [35–37] and the comparison of bioinformatics pipelines carried out by Marizzoni et al. [38] led them to conclude that “the field would likely benefit from working as much as possible with open-source, collaborative pipelines and frameworks such as QIIME2, which integrates and is continuously updated with state-of-the-art methods developed in the field”. Another major advantage of the QIIME2 platform is the fact that it does not impose a frozen workflow. Instead, several commands relying on different strategies and algorithms are available at each step of the bioinformatics analysis.

QIIME2 has initially been developed to analyze microbiome data. In consequence, several pre-formatted databases dedicated to rRNA genes (for bacteria) and the ITS region (for fungi) are directly available to carry out the taxonomic analysis of microbial HTS data. However, curated reference databases are currently lacking for other barcode sequences, which prevents taking advantage of QIIME2 features to analyze sample composition in other domains of life such as plants. In addition, even though sparse information can be found on the QIIME2 forum about how reference data should look like to be compatible with the platform, there is no detailed procedure explaining from start to finish how to develop a custom reference database for a new barcode sequence. To answer this problem, the QIIME2 development team has recently released a new plugin called RESCRIPt, to create, manage and curate reference databases [39]. Among the set of useful commands included in this plugin, the get-ncbi-data function is of particular interest as it enables retrieving from the National Center for Biotechnology Information (NCBI) repository a custom set of QIIME2-formatted nucleotide sequences, together with the associated taxonomy information. This command is thus an interesting way to create custom reference databases in an automated and straightforward manner. However, our experience has proved that this command is useful only for small sets of data. When aiming at developing a complete reference database dedicated to a barcode sequence for a whole kingdom like *Viridiplantae*, the RESCRIPt command often crashes due to the large volume of data to be downloaded, especially when dealing with chloroplastic barcodes such as *rbcL*. Indeed, some records returned from the query search are actually complete chloroplast genomes in this case, which significantly increases the volume of data to be downloaded.

More generally, users can face several bottlenecks when developing a reference database with existing pipelines. First, there is a lack of a modular/flexible workflow where the choice is left to the user whether or not to include several sequence processing steps in the pipeline. This may be particularly useful for steps like dereplication or amplicon restriction that can be relevant or not, according to the user study specifications. Also, there is a need for a workflow taking into account the fact that some reference sequences might display wrong taxonomic labels and should be filtered out. This can originate, especially for ITS plant barcodes, from environmental samples where a sequence of co-occurring fungi has been amplified instead of that of the targeted plant species. Wrong taxonomic labels can also reflect simpler cases where a plant species has been identified instead of another one. Finally, as the metabarcoding approach is becoming more and more popular, a number of research laboratories are taking advantage of this approach to perform ecology studies but sometimes without extensive bioinformatics knowledge. In this kind of situation, a detailed workflow with comments and/or advice for each command used in the pipeline would be of great help.

In consequence, this work has been set up in order to address the above bottlenecks. In addition, the aim was also to provide pre-formatted plant ITS2 and *rbcL* reference databases directly usable in QIIME2 – or in other bioinformatics platforms – to carry out taxonomic analyses. The prediction accuracy of databases developed with DB4Q2 has been assessed and compared to those of previously published databases.

## Results

### Main characteristics of the DB4Q2 workflow

**Fig. 1:**
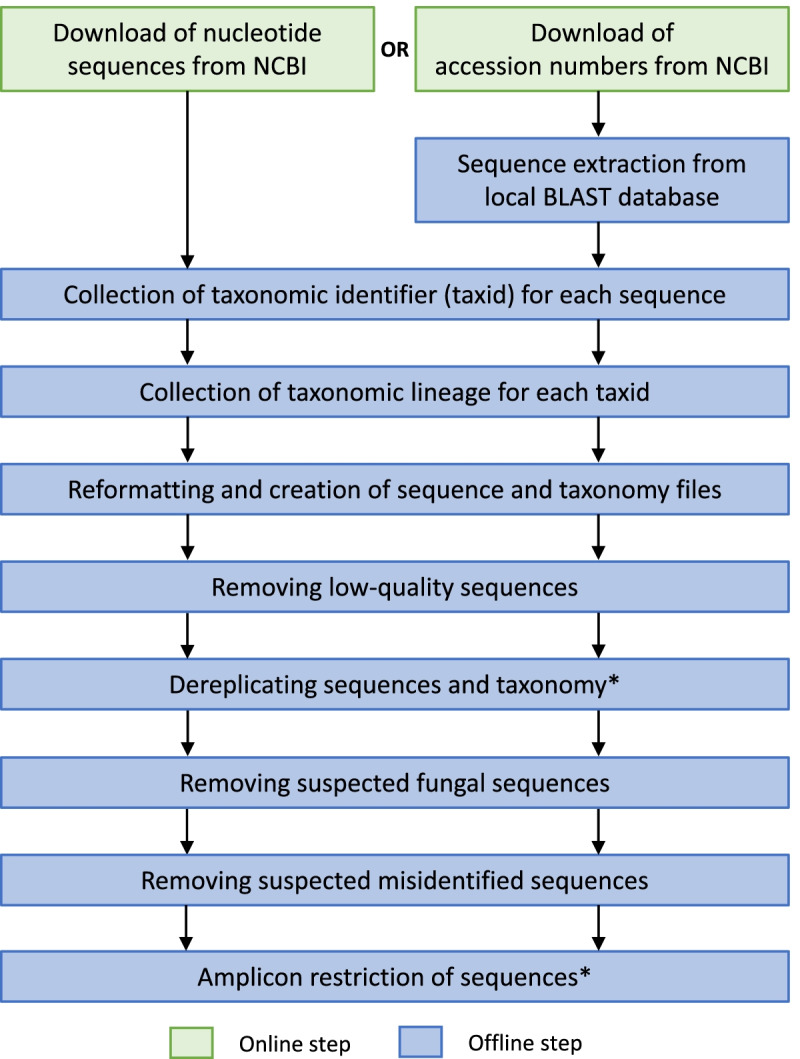
Flowchart representing the major steps of DB4Q2 to develop reference databases. Sequences can be directly downloaded from the NCBI website or extracted offline from the local nt BLAST database after having downloaded the list of sequence accession numbers. *Optional steps, the choice is left to the user whether or not to include them in the workflow

The major steps of DB4Q2 (Databases for QIIME2), the workflow presented in this work to develop reference databases, are synthetized in Fig. 1. The pipeline allows retrieving sequence and taxonomy data from the NCBI, reformatting and curating the database thanks to three quality filters: the first one removes low-quality sequences, the second one discards suspected fungal sequences and the last one filters out suspected misidentified sequences. Two optional steps allow the dereplication and the amplicon restriction of reference sequences. The choice of including these steps in the workflow is left to the user, according to its applications.

### Development of plant ITS2 and *rbcL* reference databases

The query searches carried out to collect ITS2 and *rbcL* nucleotide sequences from NCBI provided a large number of records with 238,018 and 201,740 sequences retrieved for ITS2 and *rbcL*, respectively. Even though several filtering steps were applied during the database development, it still resulted in a significant number of reference sequences and represented species (Table 1). In addition, more species were represented by the ITS2 sequence barcode compared to the *rbcL* one. For both barcode sequences, it was interesting to note that the amplicon-restricted database showed significantly fewer reference sequences than the global one, despite having set during the database restriction a similarity tolerance threshold of 0.8 between primers and reference sequences (Table 1 location).

**Table 1:** Number of nucleotide sequences and represented species in the developed plant ITS2 and *rbcL* databases at several key points of the DB4Q2 workflow

	ITS2		*rbcL*	
	Without dereplication	With dereplication	Without dereplication	With dereplication
After download from NCBI	238,018 (74,411)	238,018 (74,411)	201,740 (62,314)	201,740 (62,314)
After culling (and dereplication)	223,947 (70,339)	173,597 (70,339)	197,071 (60,769)	135,473 (60,769)
After misidentification filtering	221,954 (69,799)	171,754 (69,785)	195,946 (60,342)	134,321 (60,315)
After amplicon-based restriction	35,505 (15,425)	29,545 (15,416)	113,526 (44,269)	81,415 (44,244)

Numbers in brackets reflect the count of represented species at each step

### Comparing the databases developed in this work to previously published ones

**Fig. 2:**
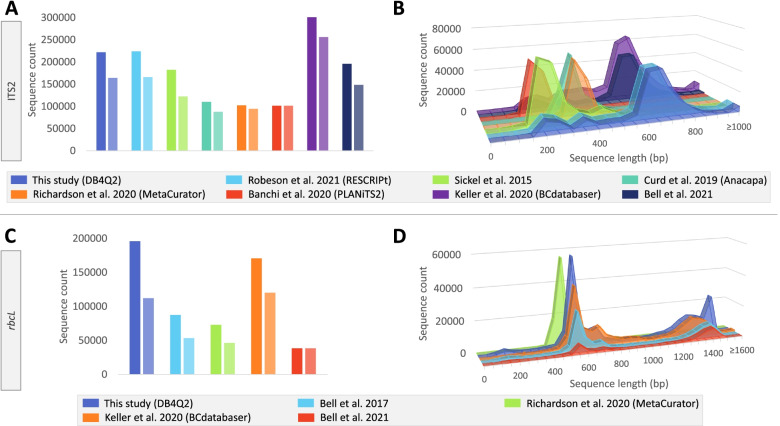
Comparison of sequence information from ITS2 and *rbcL* databases developed in this work and from previous studies. Total and unique sequence count is plotted for every ITS2 (**A**) and *rbcL* (**C**) databases included in the comparisons. For each study, the count of total and unique sequences are represented in dark and light color, respectively. The sequence length distributions are presented for every ITS2 (**B**) and *rbcL* (**D**) databases. The names of the workflows developed by the different authors are indicated in brackets. As Robeson et al. [39] did not develop an ITS2 database in their work, a reference dataset was generated with the RESCRIPt pipeline in the present study

In addition to the databases developed using the DB4Q2 workflow, an ITS2 database was generated in an automated way using the RESCRIPt plugin [39]. The same was, however, not possible for the *rbcL* barcode. Indeed, given that *rbcL* is a chloroplastic gene, many entries retrieved from NCBI after the query search were actually entire chloroplast genomes. This significantly increased the amount of data to be downloaded, which prevented using RESCRIPt to download and format data in an automated way (the ‘get-ncbi-data’ command systematically crashing despite many attempts). Ten reference datasets dedicated to the ITS2 [17, 22–26] or the *rbcL* barcodes [18, 22, 24, 26] were also identified in the literature and included in these comparisons. Analyzing sequence counts showed that databases dedicated to ITS2 held in general more sequences than *rbcL* databases (Fig. 2A and C). While large differences were observed in the number of total and unique sequences for some datasets, others exhibited (almost) identical sequence counts, reflecting their dereplicated status.

The analysis of sequence length distribution of ITS2 databases highlighted three different profiles (Fig. 2B): (i) sequence distributions from Banchi et al. [25] and Sickel et al. [17] were centered on 200 bp, (ii) the workflows developed by Curd et al. [23] and Richardson et al. [24] led to sequence datasets spanning mainly the 300–400 bp region, and (iii) the reference libraries generated in Keller et al. [26], Bell et al. [22] and in this work displayed more spread-out distributions with a peak around 700 bp. On the *rbcL* side, the database developed by Richardson and colleagues [24] was the only one where the workflow included an amplicon-extraction step and it was clearly set apart from the others, with only a single peak in its length distribution around 500 bp. In contrast, other databases showed more spread out distributions (Fig. 2D).

**Fig. 3:**
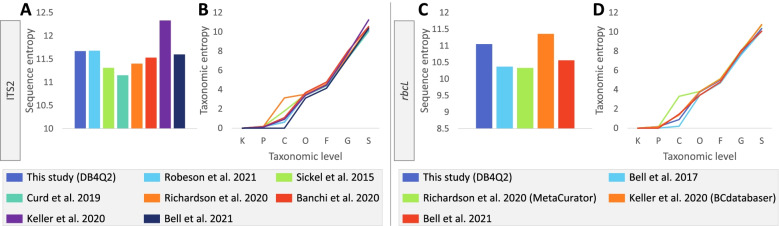
Comparison of sequence and taxonomic entropy in ITS2 and *rbcL* databases developed in this work and from previous studies. **A** Sequence entropy in ITS2 databases; **B** Taxonomic entropy in ITS2 databases; **C** Sequence entropy in *rbcL* databases; **D** Taxonomic entropy in *rbcL* databases. Rank labels on x-axis for taxonomic entropy plots: K = kingdom, P = phylum, C = class, O = order, F = family, G = genus, S = species. NB: for ITS2 taxonomic entropies, lines for databases developed using DB4Q2 and RESCRIPt are perfectly superposed

The measurement of the sequence entropy allowed evaluation of the richness of reference sequences composing each dataset (Fig. 3A and C). For both barcodes, the databases generated by the BCdatabaser workflow were outliers in these comparisons, exhibiting very high sequence entropies. A deeper analysis revealed that a part of their records was not ITS2 nor *rbcL* sequences (see details below). Besides these databases, the reference libraries developed in the present work displayed the highest entropy values, indicating that a higher sequence space is covered. The slightly higher entropy observed for the RESCRIPt database reflects the absence of filtering steps to discard suspected misidentified sequences, which removed a few thousands sequences in the DB4Q2 workflow (Table 1).

Analyzing the entropy at the taxonomy level allowed evaluation of the amount of taxonomic information held in each database at each rank (Fig. 3B and D). Here, the entropy profiles were much more similar among databases compared to sequence entropy analysis. The only major differences were observed for class labels, the taxonomic lineages displaying at this rank significantly higher and lower entropies in the databases from Richardson et al. [24] and Bell et al. [18, 22], respectively.

**Fig. 4:**
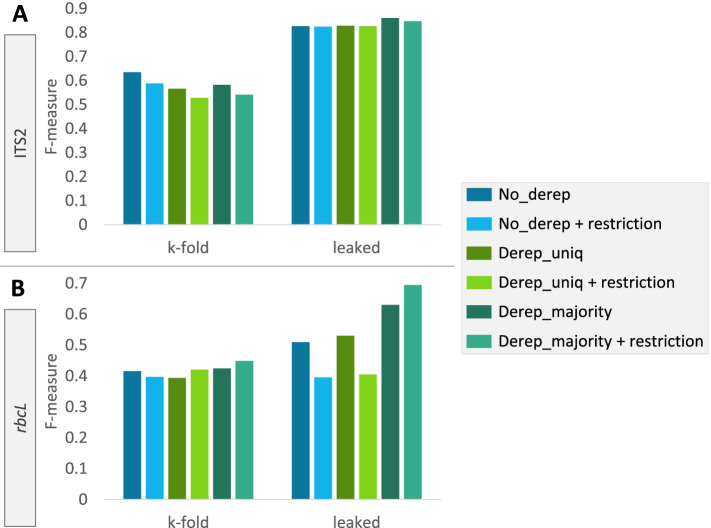
Comparison of database classification accuracy at the species level according to different dereplication and amplicon restriction settings. Prediction accuracies are presented as F-measures for the ITS2 (**A**) and *rbcL* (**B**) databases developed using DB4Q2. Accuracy scores were computed by carrying out CV tests in pseudo-realistic (k-fold) and ideal (leaked) situations. No_derep: without sequence dereplication; Derep_uniq: dereplication in ‘uniq’ mode, i.e. where identical sequences displaying different taxonomies are all conserved with their respective taxonomic labels; derep_majority: dereplication in ‘majority’ mode, i.e. where only one sequence is retained from identical sequences displaying different taxonomies, together with the most abundant taxonomic label associated with these sequences; Restriction: database amplicon restriction by extracting from reference sequences the portion amplified by a specific primer set. The dereplication in ‘majority’ mode has been tested here but is not advised nor proposed in the DB4Q2 workflow, at least for rbcL, as it can lead to a higher proportion of mislabeled sequences after dereplication

**Fig. 5:**
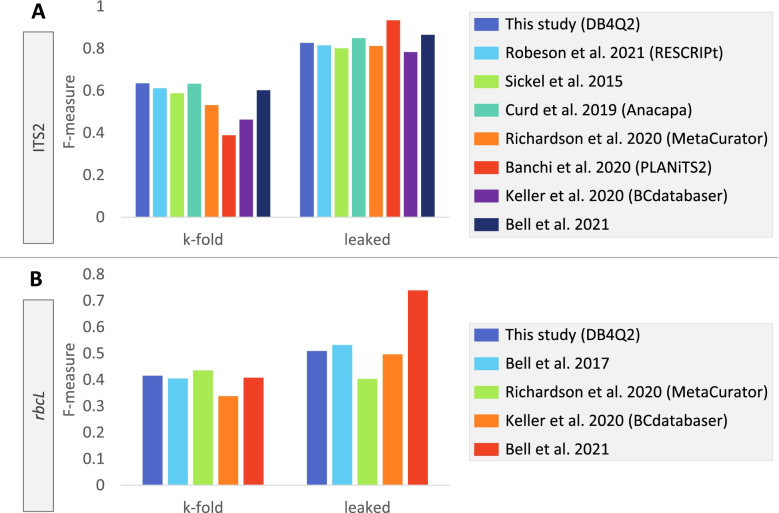
Comparison of classification accuracy at the species level from ITS2 and *rbcL* databases developed in this work and from previous studies. The comparison of classification accuracy is presented for ITS2 (**A**) and *rbcL* (**B**) databases. The pseudo-realistic classification accuracy (i.e. when subsets of reference sequences are blasted against the remaining ones and may thus not have an exact match in the training database) has been computed using a k-fold CV approach. The best possible classification accuracy (i.e. when all reference sequences are blasted against themselves and have thus an exact match in the training database) has been calculated using a leaked CV approach

In the last comparison step, the sequences in each database were classified to evaluate the classification accuracy (Figs. 4 and 5). The query sequences consisted of either the whole set of reference sequences that were classified against themselves to simulate best possible classification accuracy (designated as ‘leaked’ cross-validation (CV) to symbolize the leakage of data from query to training sequences), or only a subset of sequences that were classified against the remaining ones in a k-fold CV approach (designated as ‘k-fold’ CV). For the sake of clarity, results of these comparisons are presented below at the species rank, which is the taxonomic level where differences in accuracy scores are the most marked between databases. In addition, this is probably the level that interests the user the most in the framework of metabarcoding analyses. The complete results of these benchmarking analyses are reported for the seven taxonomic ranks in Additional files 1 (ITS2) and 2 (*rbcL*).

All databases included in this benchmarking analysis were dedicated to the *Viridiplantae* kingdom, except those developed for ITS2 by Bell et al. in 2021 [22] (*Magnoliopsida*) and for *rbcL* by Bell et al. in 2017 [18] and 2021 [22] (*Spermatophyta*). To check whether these different taxonomic breadths had a significant impact on the computed accuracy levels, *Viridiplantae* databases underwent new k-fold and leaked CV after having been restricted to the *Spermatophyta* or the *Magnoliopsida* ranks (Additional file 3). Given that no significant fluctuation could be highlighted for any of the databases when restricting the taxonomic breadth, it was decided to carry out further analyses with databases in their initial (i.e. published) status.

Dereplication and amplicon restriction of reference sequences are two steps with a significant impact on the properties of the developed database. To evaluate their influence on computed accuracies, new comparisons were carried out in pseudo realistic (k-fold) and ideal (leaked) situations with or without dereplication and amplicon restriction (Fig. 4). The dereplication was performed in two different modes: either ‘uniq’ (two identical sequences with different taxonomies are both kept and their taxonomic labels are not modified) or ‘majority’ (when identical sequences have different taxonomies, only one is retained together with the most common taxonomic label associated with these sequences). Interestingly, dereplication and amplicon restriction of reference sequences did not have the same effect on ITS2 and *rbcL* databases. Whereas these processing steps had no effect in leaked CV and even decreased prediction accuracies in k-fold CV for ITS2, the trends were different for the *rbcL* barcode sequence. Indeed, dereplicating sequences seemed to have a positive effect in leaked CV whereas amplicon restriction lowered the prediction accuracy except when associated with the ‘majority’ dereplication mode where the F-measure showed a marked increase.

Comparing databases developed in this work to those previously published showed that DB4Q2 databases were among the best performing ones, regardless of the barcode sequence or the kind of CV (Fig. 5). As already observed in previous figures, the k-fold CV showed lower F-measure values than in leaked CV, reflecting the absence of perfect match in the database queried. While some databases showed inconsistencies between k-fold and leaked CV, others displayed stable performances across conditions like those developed using DB4Q2 or Anacapa. The *rbcL* database developed by Bell et al. in 2021 showed a surprisingly high accuracy score in leaked CV, which must probably be linked to how data was processed to develop this reference dataset (see below).

## Discussion

### General workflow to develop new reference databases

In this study, we present DB4Q2, a set of detailed procedures to develop reference databases directly usable in the QIIME2 bioinformatics platform. To our knowledge, it is the first time that a detailed protocol explains from start to finish how to use a NCBI sequence dataset to develop such a database. Interestingly, this procedure can be applied for any dataset imported from NCBI, given their data structure uniformity. This means that the methodology presented can be applied to develop reference databases for any domain of life and not only for plants. In addition, it has been shown that some inconsistencies may be encountered while working with reference sequences directly imported from public databases [40]. In such cases, curating a subset of these sequences to develop a custom database is necessary and the workflow presented here should be of great help.

### Newly developed plant ITS2 and *rbcL* reference databases

After having collected and formatted all necessary sequence and taxonomic information for the sequence barcode of interest, several filtering steps are applied in order to curate the database. Two of them, dereplication and amplicon restriction, are optional and lead to major drops in the sequence count (Table 1). When carrying out dereplication, only strictly identical sequences were clustered together. Several tens of thousands of sequences were thus discarded but the amount of represented species remained the same. This reflects the ‘uniq’ mode used during dereplication, which allowed keeping identical sequences with different taxonomic labels. This step enabled discarding of redundant information and to propose more computationally efficient databases. The second optional step involved the restriction of reference sequences to the portion amplified by commonly used PCR primers. This had a strong impact on the count of sequences and represented species in databases. The utility and relevance of these two optional steps are discussed below.

After having applied all filtering steps, a little more than 60,000 species were represented in the *rbcL* reference databases, reflecting a marked increase compared to the 38,409 plant species reported by Bell et al. in 2017 [18] and to the 49,936 species in the database developed in 2020 by Richardson et al. [24]. For the ITS2 sequence barcode, the almost 70,000 species represented in the databases also illustrated an increase in species count compared to the 54,164 plant species reported by Richardson et al. [24]. It is, however, a little less than the 72,325 species reported by Sickel et al. [17], which reflects the effect of the different filters applied in DB4Q2 and not present in the workflow of Sickel and colleagues.

### Addressing existing bottlenecks when developing a new reference database

As previously mentioned, some ITS2 and *rbcL* reference databases have already been published but, for some of them, without precise explanations detailing how reference datasets were generated. Such a ‘black box’ system should be avoided in order to have a clear visibility on each step of the workflow. That is the reason why DB4Q2 has been extensively detailed and commented, so that the user can understand which operation is carried out at each step and evaluate the relevance according to its study specifications. Furthermore, with the advent of HTS technologies, many laboratories are launching new research activities using DNA metabarcoding but sometimes without extensive bioinformatics knowledge. In this kind of situation, it is not rare to see the use of existing tools in a rather blind manner or the outsourcing of analyses, which lowers the control and understanding that the user has on every database-processing step. The detailed procedures presented in DB4Q2 should also help those teams avoid this kind of problems.

When evaluating how current bottlenecks are addressed with DB4Q2, it is interesting to compare it with RESCRIPt since they are both intended to generate QIIME2-formatted databases. RESCRIPt is a remarkable tool built by the QIIME2 developer team with many useful applications. However, we noticed that the command used to import directly from the NCBI a reference dataset and format it in an automated way into a functional database could not handle large datasets, probably due to NCBI download limitations. This issue was faced when trying to retrieve the *rbcL* reference dataset and should probably occur often when dealing with other plant chloroplastic barcodes (or with mitochondrial barcodes commonly used e.g. in animal metabarcoding). Indeed, a part of the entries downloaded from the NCBI is actually complete chloroplast/mitochondrion genomes, which significantly increases the volume of data. The DB4Q2 provides an answer to this bottleneck since it allowed downloading both ITS2 and *rbcL* datasets without any issue. In addition, our workflow also proposes an almost completely offline procedure to skip this downloading step and associated difficulties.

Another bottleneck the user may face when developing a reference database is the inaccuracies of taxonomic identifications in NCBI records [41–43]. This sequence mislabeling can of course hinder accurate taxonomic assignment of sequencing reads but also lead to perpetuation of errors when using these data [44]. Given that fungi are often co-occurring in surface or inside plant tissues, this issue is particularly true in plant metabarcoding studies. Indeed, there is an additional risk of amplifying fungi DNA instead of, or together with, that of the target plant species [45], especially when working with ITS primers, even if they are plant-specific [46]. Beside this problem related to fungi sequences, a reference sequence may simply have been assigned to a plant taxa instead of another one. To remove these entries, blasting all database sequences against themselves allowed discarding those for which the expected taxonomy at the family rank was observed only once in the five best matches. This strategy should allow filtering out many misidentified entries but probably not all. Indeed, the comparison of expected and predicted taxonomies could not be carried out at a lower taxonomic rank since the exact same sequence can be shared by several species and even sometimes several genera when a barcode marker does not display enough sequence divergence. Hence, if expected and predicted taxonomies were compared at the genus or species level, the risk would be to discard sequences for which the identification was actually correct. This is the reason why we chose the family rank as an appropriate trade-off between filtering out enough mislabeled sequences while avoiding as much as possible the removal of sequences correctly identified. To evaluate the impact of these filters, it is interesting to note that the first parts of the DB4Q2 and RESCRIPt workflows are almost identical but there is no filter to remove fungi sequences nor more generally mislabeled plant sequences in RESCRIPt. The increase in prediction accuracy observed between RESCRIPt and DB4Q2 databases (Fig. 5) thus provides a good illustration of the beneficial effect of these filtering steps.

Among previously published reference databases and pipelines, several strategies are observed like the use of trimmed reference sequences provided by the user to build an amplicon-restricted dataset [24], querying public repositories using user-defined primers [23], the sequence dereplication taking their taxonomy into account [24] or even their clustering at 99% identity [25]. Despite being very interesting, these strategies may not be relevant for every research context. For example, it has been shown that the amplicon restriction of reference sequences can have a positive impact on taxonomic predictions for some barcode sequences [47], whereas it is not recommended for others [48]. In consequence, DB4Q2 has been written with some optional sections so that the user can decide whether or not to include these critical steps in the workflow.

### The importance of (not) dereplicating database

Dereplication is a sequence-processing step commonly used to build a curated reference database [24, 25, 39]. It often allows a significant reduction of the database size, thus increasing its computational efficiency. When analyzing metabarcoding data, some widely used taxonomic classifiers are based on a consensus strategy by considering the taxonomic labels of e.g. the five or ten best matches from the database to assess the taxonomy of sequencing reads. Considering that, the dereplication step presents the additional advantage to give more weight to under-represented taxa in the database. On the counterpart, more frequent taxa are thus disadvantaged in such an approach by setting them on equal footing with under-represented ones, which is probably not the best strategy when working in deeply studied areas.

The most relevant dereplication approaches take taxonomic labels into account to discard identical sequences. In this work, the influence of this step was tested according to two dereplication settings. The first one is the ‘uniq’ mode, where two identical sequences with different taxonomies are both kept and their taxonomic labels remain unchanged. In the second mode (‘majority’), when identical sequences have different taxonomies, only one is retained together with the most common taxonomic label associated with these sequences. In k-fold CV tests, sequence dereplication did not have a significant impact for the *rbcL* barcode while it tended to decrease the prediction accuracy for ITS2 (Fig. 4). Conversely, in leaked CV tests, it did not have a major effect for the ITS2 databases while *rbcL* accuracy values seemed to be positively affected, especially in majority mode. This other example illustrates the effect that some parameter choices can have on metabarcoding analysis outcome and thus supports the flexibility of DB4Q2 with its optional sections, including at the dereplication step.

It must however be noted that the dereplication in ‘majority’ mode has been tested but is not advised nor proposed in the DB4Q2 workflow, at least for *rbcL*, as it can lead to a higher proportion of mislabeled sequences after dereplication. Despite the fact that relabeling of identical sequences with the most frequent taxonomic lineage can be seen as a convenient way to correct identification mistakes, it must be avoided when working with barcodes with insufficient sequence divergence (like *rbcL*). Indeed, it is not rare to face several species that have the exact same *rbcL* sequence and relabeling all them with the most frequent taxonomy would erroneously increase computed prediction accuracies (as observed for *rbcL* in Fig. 4) while not being representative of the taxonomic diversity anymore.

### Comparison with other published databases

The ITS2 and *rbcL* reference databases developed in this work were compared to the published ones presented above. These comparisons allowed investigation of the sequence and taxonomic information held in each database, as well as evaluating the accuracy of their taxonomic assignments (Figs. 2, 3 and 5).

For both barcode sequences, the DB4Q2 databases showed the highest unique sequence count compared to other databases (Fig. 2A and C). This can be explained by their recentness compared to others. In addition, they did not undergo an amplicon extraction – which unavoidably provokes a sequence loss – while the databases developed in Curd et al. [23] and Richardson et al. [24] did. The only exception is the ITS2 and *rbcL* datasets built with BCdatabaser [26], which displayed a surprisingly high number of sequences, particularly for the ITS2 barcode. A deeper analysis showed that a part of the sequences in the database did not cover the ITS2 region (e.g. more than 27,000 sequences displayed the string “external transcribed spacer” in their definition line). This means that the query string inserted in the pipeline probably matched with more than only ITS2 sequences. A similar observation was made with the *rbcL* database for which almost 13,000 sequences did not exhibit the keywords “*rbcL*” or “ribulose” in their definition line (but they did in the article title section of their Genbank record for example, which could explain the confusion). These observations explain the higher peaks observed for BCdatabaser datasets in Fig. 2A and C. The databases developed by Banchi et al. for ITS2 [25] and Bell et al. in 2021 for *rbcL* [22] are dereplicated and thus showed identical counts for total and unique sequences.

The comparison of sequence length distribution showed that ITS2 sequences were on average shorter than *rbcL* ones (Fig. 2B and D). This is consistent with the fact that the ITS2 fragment is in the 200–250 bp range [49, 50] while the *rbcL* gene is about 1400 bp long [51]. This comparison also revealed a close relationship between the strategy used to generate these databases and their length distribution profile. For ITS2, Sickel et al. [17] and Banchi et al. [25] used hidden Markov models to extract only the ITS2 portion from downloaded sequences, which explains the shorter average length for these datasets. The databases developed by Richardson et al. [24] and Curd et al. [23] exhibited sequence length distribution centered around 300–400 bp and thus reflected the sequence amplicon extraction carried out in both workflows. The last group of ITS2 reference libraries included the ones developed by Keller et al. [26], Bell et al. [22] and those built using RESCRIPt [39] and DB4Q2. No sequence extraction step was performed in any of these studies, which explains why their length distribution profiles are more spread out. The peaks observed for these databases around 700 bp reflect mostly cases where the amplicon spanned the ITS1–5.8S-ITS2 region. On the *rbcL* side, besides the individual peak observed for the amplicon-restricted database from MetaCurator, the peaks visible around 600 bp and 1400 bp correspond respectively to the typical length of barcode markers used in Sanger sequencing on the one hand, and to the complete sequence of the *rbcL*-coding gene on the other hand.

Interestingly, when investigating sequence entropy, the databases developed using DB4Q2 compared well to published databases for both barcodes, despite having discarded several thousand sequences that did not meet quality requirements (Fig. 3A and C). This high sequence entropy can be attributed to several factors like the database recentness, the absence of an amplicon extraction step and the taxonomic coverage of downloaded sequences: most databases studied here cover the whole kingdom of plants whereas Bell et al. developed *rbcL* and ITS2 databases dedicated to the *Spermatophyta* clade (seed plants) and the *Magnoliopsida* class (flowering plants), respectively. The higher entropic values observed for BCdatabaser reference libraries must be analyzed with caution given that a fraction of their records are actually not ITS2 nor *rbcL* sequences, as previously mentioned.

To evaluate the amount of information at each taxonomic rank in each database, the taxonomic entropy was measured (Fig. 3B and D). The greater variability observed at the class level reflects two distinct phenomena. On the one hand, the databases from Bell et al. with restrained taxonomic breadth (see above) explain the lower class-level taxonomic entropies observed for these databases. On the other hand, it was noticed that several databases included in this comparison did not display any class label in their taxonomic lineages, and this problem occurred mostly for the *Manoliopsida* class. Instead, the labels showed annotations related to lower taxonomic ranks like ‘c__urs_o__Brassicales’ or ‘c__sub__asterids’. This significantly increased the amount of information in class-level labels, which led to an overestimation of the taxonomic entropy at this rank. The trends were much more similar between databases for the other taxonomic levels, BCdatabaser slightly differing from the other workflows at the species rank for the reasons mentioned earlier.

Finally, the classification accuracy of ITS2 and *rbcL* reference databases was evaluated (Fig. 5). In general, the *rbcL* databases showed lower accuracy scores compared to ITS2 datasets. This reflects the higher degree of conservation of the *rbcL* barcode compared to ITS2, as *rbcL* may not display enough sequence divergence across taxa, even sometimes at the genus level.

Classifying all sequences against themselves (leaked CV) revealed that the reference datasets generated in this work were among the ones with the highest scores at the species rank. Notably, the *rbcL* reference library developed by Bell et al. in 2021 [22] exhibited a significantly better classification accuracy. To build this database, the authors appended new reference sequences from Australian plant species to the one built in 2017. Despite the addition of new sequences, this updated database displayed fewer sequences (with identical counts of total and unique sequences) and an identical taxonomic entropy compared to the one built in 2017. Given that the authors mentioned further formatting work, the most probable hypothesis is that a sequence dereplication in majority mode has been carried out, which is known to increase prediction accuracy in leaked CV (Fig. 4). In addition, the lower amount of sequences in this dataset also favors better accuracy scores. Indeed, the fewer sequences are included in the comparison, the lower is the risk that the classification accuracy is confounded by other similar hits.

When assessing taxonomic accuracy in a k-fold CV scheme, the dereplicated databases should theoretically be disadvantaged. Indeed, in this case, it is less likely that a second sequence belonging to a particular taxon is present in the training dataset if a first one has already been extracted to be included in the test set. The comparison of ITS2 reference datasets could indeed highlight this phenomenon, the dereplicated databases exhibiting F-measures slightly (MetaCurator) or strongly (PLANiTS2) decreased compared to the best accuracy levels measured (Fig. 5A).

Interestingly, the comparison of results obtained in best possible (leaked CV) vs pseudo-realistic conditions (k-fold CV) highlighted very contrasting performances for databases developed by Banchi et al. (ITS2) and Richardson et al. (*rbcL*). This indicates that, according to the kind of samples analyzed and their expected level of challenge (i.e. composed of taxa either with or without existing reference sequences), these databases will probably show very different classification accuracies. In contrast, other reference datasets including the ITS2 and *rbcL* databases developed using DB4Q2 displayed consistent accuracy levels in both situations, making them versatile reference datasets to be used in DNA metabarcording analyses.

## Conclusions

In this work, we have developed the DB4Q2 bioinformatics workflow and constructed plant reference databases dedicated to the ITS2 and *rbcL* barcode sequences. These databases were formatted to be used in QIIME2, one of the most commonly used bioinformatics platforms to carry out DNA metabarcoding analyses. In addition, every file corresponding to reference sequences and associated taxonomies are also provided in a standard format, to offer the possibility of using these databases in other bioinformatics platforms if desired. Benchmarking the performances of these databases highlighted that they performed well in comparison with previously published reference databases. Notably, the workflow developed is presented in a standardized procedure in such a way that it can be applied to develop reference databases for any barcode sequence and any domain of life.

## Methods

### The DB4Q2 workflow

The detailed and commented DB4Q2 workflow to develop a QIIME2-formatted reference database is available on our figshare repository (10.6084/m9.figshare.17040680), together with examples of developed plant reference databases (see below for details). As a preamble, it must be noted that the scripts have been written to develop QIIME2-formatted reference databases (qza files) so that they can be directly used in this platform to carry out taxonomic analyses. However, these databases can also be used on other bioinformatics platforms if desired; fasta files (for reference sequences) and tsv files (for reference taxonomies) are thus provided in the figshare repository, in addition to QIIME2-formatted files.

#### Development of a plant ITS2 reference database

The workflow to develop a plant ITS2 reference database is made of ten main parts. (i) The first step consisted of collecting from the NCBI website all available plant ITS2 nucleotide sequences (as of 9 August 2021), using the query search: ((viridiplantae[Organism] AND its2) AND 100:10000000[Sequence Length]) NOT (uncultured OR environmental sample OR incertae sedis OR unverified). The script also presents an almost completely offline method as an alternative way to retrieve this nucleotide sequence dataset. In this ‘offline’ alternative, the only step requiring an internet connection is the download of accession numbers from the NCBI website. This list is then used offline to extract the corresponding nucleotide sequences from the local nt NCBI BLAST database. (ii) The second step involved, for each nucleotide sequence, the retrieval of the taxonomic identifier (taxid) of the organism the sequence comes from. This was carried out using the “nucl_gb.accession2taxid” file available on the NCBI ftp website. Even though this problem was not faced for this database, experience has shown us that several taxids may not be retrieved at the end of this step. In consequence, the script also shows how to build an API query to the NCBI Entrez system to recover missing taxids, if any. (iii) Once taxids were recovered, the associated taxonomic information has been sought for in the “new_taxdump” file available on NCBI ftp website. Seven-level taxonomic lineages were created by extracting, for every taxid, the information for the kingdom, phylum, class, order, family, genus and species taxonomic ranks. Again, if some taxonomic lineages happened to be missing at the end of this step, the script offers a complementary way to recover them. (iv) A global table was then generated to gather, for each database entry, the accession number, the nucleotide sequence, the taxid and the taxonomic lineage. (v) This global table enabled creation of two files (corresponding to fasta-formatted nucleotide sequences and their corresponding taxonomy), which were then imported into QIIME2. (vi) These files were then processed to discard sequences displaying ≥5 degenerate bases or containing a homopolymer sequence of ≥12 nucleotides. (vii) The next step is optional and involves a sequence-taxonomy dereplication to remove redundant data and deal with identical sequences displaying different taxonomies. As the relevance of such dereplication depends on several factors including the barcode of interest, the choice is left to the user to include it or not in the workflow. (viii) Sequences suspected to be mislabeled and to belong to fungi were removed by blasting reference sequences first against fungi genomic RefSeqs and then against fungi ITS sequences from the UNITE database. After both blastn analyses, sequences displaying at least 90% similarity on at least 95% of their length with fungi sequences were discarded. (ix) To ensure the correctness of taxonomic labels, another filtering step was then applied to remove sequences suspected to have a wrong identification. To do so, ITS2 reference sequences were blasted against themselves and expected and predicted taxonomies were compared for each sequence. This allowed discarding those for which the expected taxonomy at the family rank was observed only once in the five best matches resulting from the blastn analysis. The resulting curated files can then be used inside (or outside) QIIME2 and an additional step has also been added to pre-train a classifier if taxonomic analyses are planned to be carried out with the sklearn-based Naive Bayes approach. (x) The last step allows restricting reference sequences to only the portion sequenced. As the relevance of this strategy also depends on the barcode sequence, the choice of including this step in the workflow is left to the user.

#### Development of a plant *rbcL* reference database

The detailed script presenting the development of a QIIME2-formatted plant *rbcL* database is available on our figshare repository. Both database files (sequence fasta file and taxonomy tsv file) can also be obtained on the repository to use this reference database outside of QIIME2, if desired. All available plant *rbcL* sequences were retrieved from NCBI on 14 June 2021 with the following query: (viridiplantae[Organism] AND (*rbcL*[Gene Name] OR ribulose-1,5-bisphosphate carboxylase/oxygenase[Title] OR ribulose-1,5-bisphosphate carboxylase oxygenase[Title] OR *rbcL*[Title]) AND 100:500000[Sequence Length]) NOT (uncultured OR environmental sample OR incertae sedis OR unverified). After that, most of the procedure was identical to the one detailed previously in the development of the plant ITS2 reference database. The major difference relies in the chloroplastic origin of the *rbcL* gene, in contrast to the nuclear ITS2 sequence. This implies that some of the records matching with this query search are actually complete chloroplast genomes. In consequence, nucleotide sequences were downloaded in the “Gene Features” NCBI format in order to selectively extract *rbcL*-coding gene sequences from chloroplast genomes. In addition, the fungi sequence-filtering step is carried out by blasting *rbcL* sequences only against fungi genomic RefSeqs since using UNITE ITS sequences would be irrelevant in this case.

### Database benchmarking

To place the developed databases in the context of previous works and compare performance across methods, ITS2 and *rbcL* reference datasets published in other studies were downloaded to compare them all together. In total, six ITS2 databases [17, 22–26] and four *rbcL* databases [18, 22, 24, 26] were downloaded. These datasets were imported into QIIME2 in their published status, only minor formatting changes being applied (e.g. addition of prefixes to taxonomic ranks when necessary). In addition, a plant ITS2 database was also created using the ‘get-ncbi-data’ command of RESCRIPt [39]. The command was executed the same day and with the same query terms as in the DB4Q2 workflow to ensure that both sequence datasets downloaded from the NCBI were strictly identical. Sequences in this database were also curated using the ‘cull-seqs’ command with same parameters as in DB4Q2. The RESCRIPt plugin could not, however, be used to build a database dedicated to the *rbcL* barcode since the dataset to be downloaded was too large and it made the ‘get-ncbi-data’ command crash.

The QIIME2 evaluate-* family of actions was used to evaluate the sequence and taxonomic information held in each database. It was also used to assess the classification accuracy by classifying the sequences in the databases. This database accuracy evaluation was performed in two ways. (i) The ‘evaluate-cross-validate’ action used k-fold cross validation to split databases into K test sets (K was set to 5), in such a way that each sequence appeared only once in each test set. Classification was then performed in each fold with the remaining sequences as the training set [52]. This strategy allowed carrying out pseudo-realistic classification, where query sequences may not have an exact match in the reference database. (ii) The ‘evaluate-fit-classifier’ action was used to test classification on the full dataset, i.e. with data leakage from the test set to the training set [39]. Each query sequence had thus an exact match in the database, which allowed simulating the best possible classification accuracy when the true label is known, knowing that the classification accuracy may still be confounded by other similar hits in the database. For these two methods evaluating classification accuracy, the precision and recall metrics were computed to take into account the proportion of false positives and false negatives, respectively. The F-measure metric, i.e. the harmonic mean of precision and recall, was then calculated to compensate for some opposite fluctuations these two parameters might exhibit [39].

## Supplementary Information

**Additional file 1.**

**Additional file 2.**

**Additional file 3.**

## Data Availability

Documentation, scripts and individual database files are available at the figshare repository: 10.6084/m9.figshare.17040680.

## Abbreviations

API: Application Programming Interface
CV: Cross-validation
DNA: Deoxyribonucleic Acid
HTS: High-throughput Sequencing
ITS: Internal Transcribed Spacer
NCBI: National Center for Biotechnology Information
PCR: Polymerase Chain Reaction
*rbcL*: ribulose-1,5-bisphosphate carboxylase-oxygenase
rRNA: Ribosomal Ribonucleic Acid
